# Evaluating Turkish society's approaches to hemorrhoid treatment using text mining and sentiment analysis methods

**DOI:** 10.3389/fsurg.2026.1927511

**Published:** 2026-09-18

**Authors:** Adem Doğaner

**Affiliations:** Department of Biostatistics and Medical Informatics, Faculty of Medicine, Kahramanmaraş Sütçü İmam University, Kahramanmaraş, Türkiye

**Keywords:** hemorrhoids, infodemiology, sentiment analysis, social media, vader

## Abstract

**Background:**

Hemorrhoids is a rectal disease that has adverse effects on life quality. Because the disease occurs in a discreet area, the rectal, patients often hesitate to consult a physician out of embarrassment. Thus, they often turn to the internet to find support and solutions. The ultimate objective of this study is to determine the societal attitudes, behaviors, and perceptions towards hemorrhoids in Turkey. To achieve this, a comparative sentiment analysis of social media comments was performed for the purpose of getting an idea about different treatment methods' effectiveness and satisfaction rate.

**Methods:**

The data set used includes user comments on YouTube videos in Turkish on the topic, which are open to public access. In this regard, totally 15.382 comments under 116 videos were analyzed. Sentiment analysis using a dictionary-based model was done, followed by the process of obtaining a sentiment score for each comment via the VADER model. The comments were divided into four groups (treatment methods) using the LDA (Latent Dirichlet Allocation) method based on the words related to their subject matter: surgical, pharmaceutical, herbal, and traditional.

**Results:**

In terms of sentiment scores regarding treatment methods (VADER), the differences among YouTube users were found to be statistically significant (KW-H = 43.873, *p* = 0.001). YouTube user comments regarding herbal treatment methods had the highest positive sentiment scores (mean ± SD = 0.069 ± 0.260). A statistical difference was found in the frequency of the term “recovery” among YouTube user comments regarding surgical treatment methods (X^2^ = 24.972, *p* = 0.001) The word “recovered” was used in 34.1% of YouTube user comments regarding surgical treatment methods. Similarly, a statistical difference was found in the frequency of the word “recovered” among YouTube user comments regarding herbal treatment methods (X^2^ = 19.985, *p* = 0.004). According to sentiment analysis, 14.7% of comments on surgical treatment methods, 16.5% of comments on traditional treatment methods, 20.9% of comments on herbal treatment methods, and 19.7% of comments on medical drug treatment methods were positive. According to the comments, while surgical methods are the most effective way to eliminate hemorrhoids, herbal, pharmaceutical, and traditional methods are understood to be effective only in alleviating symptoms.

**Conclusion:**

Turkish societal attitudes, feelings, and treatment methods preferences toward hemorrhoids were determined through sentiment analysis and text mining methods. Consequently, data obtained from social media tools provides valuable insight into societal health-related tendencies and behaviors.

## 1 Introduction

### 1.1 Hemorrhoids

The main reason for hemorrhoids is enlargement and displacement of anal cushions, which can be observed in anorectal region (1, 2). Hemorrhoids are consisted of submucosal cushions that supports the closure of the anal canal (3). Although hemorrhoids are so common, many people experience no symptoms and do not seek medical support. Different studies have been performed on diverse societies and age groups so far, but because of the mentioned situation, it has been impossible to obtain definite, non-variable results (4). Consequently, Haas et al. (5) reported that 86% of adults had hemorrhoids (5). According to Riss et al. (6), 38.93% of adults in Austria had hemorrhoids (6). Hong et al. (7) estimated that the frequency of the disease to be 16.6% (7). Both men and women are commonly affected by this disease. There are two types of hemorrhoids: internal and external (8). Internal hemorrhoids tend to cause fewer symptoms than external hemorrhoids. Based on clinical findings and structure, hemorrhoids generally progress through four stages. The disease has generally no symptoms in the early stages, but in the advanced stages the patient can have symptoms such as bleeding, itching, pain, cramping, discharge, and swelling. Hemorrhoids are often painful and uncomfortable, which can significantly reduce patients' life quality and cause the formation of various colorectal diseases (7, 9). Hemorrhoids can be treated by several methods such as surgical, non-surgical, and conservative treatments. The scheduled treatment method may also change depending on the physician's disease management strategy. In general, surgical and conservative treatment methods are preferred as the first step (10). Colorectal surgical procedures are frequently used in the treatment of hemorrhoids. Colorectal surgical procedures are generally preferred for the treatment of stage 3 and 4 hemorrhoids (11). It is well known that open hemorrhoidectomy and closed hemorrhoidectomy are the most commonly performed surgical procedures. Although there are fundamental differences between open hemorrhoidectomy and closed hemorrhoidectomy—such as postoperative pain and the amount of bleeding—the procedures are similar in terms of the overall process (12). It has been observed that open and closed hemorrhoidectomies result in slower recovery and a more painful treatment process during the postoperative period compared to stapled hemorrhoidectomy. In contrast, patients undergoing stapled hemorrhoidectomy experience a higher rate of hemorrhoid recurrence (13). Although stapled hemorrhoidectomy may seem like a more reasonable treatment option in terms of patient comfort, the high recurrence rate associated with this procedure makes it a less favorable option (14). It has been reported that complications such as postoperative rectal discomfort and chronic pain have been observed with the stapled hemorrhoidectomy technique (15). Another technique used in colorectal surgery is transanal hemorrhoidal dearterialization (THD). THD is a surgical technique that is often used for second, third, and fourth degree hemorrhoids (16). It has been observed that the THD technique yields more successful outcomes in terms of postoperative complications compared to open hemorrhoidectomy and stapled hemorrhoidectomy. However, it has been observed that the THD technique has a higher recurrence rate compared to open hemorrhoidectomy (13, 17).

When determining colorectal surgical approaches, the stage of the disease is the primary consideration. In cases where pharmacological treatment has failed, surgical options may be considered (11). In cases such as acute thrombosis and necrosis, emergency surgery may be considered (15). Although colorectal surgery is effective for advanced hemorrhoids, combination therapies may yield better outcomes in terms of complications (18).

Surgical methods are usually yield effective results in treatment. However, patients may be anxious about pain, complications, continued bleeding, and recurrence of the disease after the operation (19). These complications following the surgical treatment lead patients to look for alternative methods. Among the frequently used conservative methods patients use to ease the symptoms are dietary changes, eating habit modifications, and topical applications (20). Pharmacological treatments that reduce the severity of symptoms and manage the phases of the disease are another commonly used method. These treatments, which can be used in different forms such as oral medications, suppositories, creams, and gels, are generally used to stop bleeding, alleviate pain, and reduce swelling. Effective pharmacological treatments that reduce the severity of symptoms and manage the progression of hemorrhoid disease can be used alongside surgical methods (21). Patients also have used various methods alongside modern medical treatment methods to alleviate their symptoms and improve their condition. Acupuncture is one of them, which helps to alleviate pain after surgery (22). Traditional Chinese medicine and treatment methods, such as cupping, are widely used in Asian countries. These methods are generally used for the aim of stop bleeding and reduce symptoms (23). One of the treatment methods used for hemorrhoids is sclerotherapy. The primary goal of sclerotherapy is to destroy the endothelium in order to eliminate the veins. For this procedure, liquid or foam solutions are injected. The sclerosing agent administered causes sclerosis to develop in the vessel wall (24). In sclerotherapy for hemorrhoid treatment, a sclerosing agent is injected into the internal hemorrhoidal plexus to cause the vessels to close off and form fibrosis (25, 26). A polidocanol foam formulation is typically used in the sclerotherapy method for the treatment of hemorrhoids (27). Sclerotherapy yields successful results in the treatment of grade I–III hemorrhoids. However, for advanced-stage hemorrhoids, rather than serving as a direct treatment option, it acts as a bridge to surgical treatment (28–32). For this reason, sclerotherapy is generally recommended for patients with stage I–III hemorrhoids (33). Another method used in the treatment of hemorrhoids is herbal treatment. Although surgical treatments can completely remove hemorrhoids, herbal treatments cannot fully cure them. Herbal treatments generally aim to alleviate the symptoms of hemorrhoids in the early stages. Herbal treatments can reduce bleeding from hemorrhoids, reduce swelling, and relieve pain. However, not every plant is effective in reducing symptoms during hemorrhoid treatment (34). The number of studies examining the effects of herbal treatments on hemorrhoids in humans is quite limited. Studies indicating that herbal treatments have a therapeutic effect on hemorrhoids generally consist of animal experiments or *in vitro* research (35, 36).

The most effective treatment for hemorrhoids is colorectal surgery. Both open and closed hemorrhoidectomy procedures provide successful excision of hemorrhoids. With these methods, the recurrence rates for hemorrhoids are lower than with other methods. Meta-analyses have shown that surgical treatment is more effective than conservative treatments in resolving symptoms. Similarly, it demonstrates that the surgical method is statistically more effective than conservative methods in reducing pain (10). Pharmacological treatment methods for hemorrhoids are generally used to relieve symptoms and manage pain. Pharmacological methods are not as effective as surgical methods in treating hemorrhoids. Conventional pharmacological treatments do not provide a permanent solution for hemorrhoids (37). Due to the lack of scientific studies on the use of herbal and traditional methods for the treatment of hemorrhoids, these methods offer a low level of scientific evidence. There are studies reporting that plants such as aloe vera and horse chestnut may help alleviate postoperative pain. But the lack of randomized controlled trials on these methods undermines confidence in herbal treatments (38).

The comments on YouTube videos about hemorrhoid treatment primarily focus on differences in treatment approaches, pre- and post-treatment processes, complications, and the success of the treatment. Each comment posted by YouTube users reflects the results of the methods used in the treatment of hemorrhoids. There is a cause-and-effect relationship between hemorrhoid treatment methods and YouTube user comments. Youtube users' comments can reveal how people feel about the method used to treat hemorrhoids. Knowledge of the processes and literature regarding the various methods used in the treatment of hemorrhoids is important for assessing the consistency of sentiments expressed in the comments.

### 1.2 Sentiment analysis and text mining on social media

For common diseases which are commonly observed among the population, such as hemorrhoids, most people prefer to get information from various sources rather than directly consulting medical facilities. In recent years, number of websites and social media platforms, which provide information about various diseases have increased. Both patients and healthcare professionals can access these interactive websites to share information. One of them is YouTube. YouTube is one of the highly popular online platforms (39). YouTube is an online video site that contains a large number of content on almost every subject and allows users to share comments. Number of health-related videos on YouTube are quite high. Some of the health-related videos have been created by healthcare professionals. However, some are uploaded by non-experts and may contain misleading or incomplete information (40). On YouTube, users has the right to comment and share their thoughts, experiences, or knowledge related to the video's topic. These comments may be good sources to analyze people's thoughts, opinions, attitudes, or level of knowledge on a specific topic.

It is reasonable that comments from users on health-related videos on social media sites, like YouTube, might be beneficial for patients because they include personal experiences, recommendations, and practical treatment options. But unfortunately, this dataset is too large and most of the text contained is unprocessed (41). Methods such as text mining, which are used in both infodemiological and scientific research, can be employed to extract meaningful information, so that patients can really benefit from these data (42).

Text mining includes methods that help to automatically discover information from textual data sources. Text mining can be used with either structured or unstructured textual content. The purpose of text mining is to expose the structural relationships within text and provide meaningful information (43, 44). Thanks to the increase in social media data sources, it became possible to examine infodemiological studies using text mining methods. Infodemiological research on health-related information that is accessible via websites and social media platforms focus on ensuring that people can access to accurate, high-quality, and dependable data online (45). Data obtained from social media platforms have made it possible to conduct infodemiological assessments, such as evaluating the spread of diseases, societal attitudes and behaviors toward diseases, and also spread of misinformation. Infodemiological research has gained popularity, especially during the Covid-19 pandemic. During the Covid-19 pandemic period, infodemiological conclusions about patients' symptoms and general attitudes were obtained using data from social media platforms (46). Moon and Lee conducted an infodemics study examining how YouTube provided useful information during South Korea's rapid decline in Covid-19 cases. In their study, they observed that videos prepared by public institutions in particular provided useful information to people (47). The fact that text mining methods played an important role made it possible to assess social media data in a practical way played an important role in the expansion of infodemics research. There are different methods in text mining used to extract information from texts. One of those methods is sentiment analysis. Unlike other text mining methods, sentiment analysis includes algorithms that can process classification even in texts containing irony and metaphors (48). People using social media usually write comments with irregular and unstructured sentences, words used outside their literal meanings, and use ironic or humorous expressions. So, considering that the language on social media is structured and regular can lead to inaccurate conclusions. Sentiment analysis is highly advantageous for analyzing textual comments that include irony and metaphors, such as humorous posts. Dictionary-based models and machine learning methods can be used for sentiment analysis. Greaves et al. (49) performed a sentiment analysis of comments about patient experiences on social media sites by using machine learning methods (49). Sentiment analyses which are performed using social media data can be beneficial for understanding social behavior and thinking, as well as creating public health strategies (50). People may use social media to find information about health. Sentiment analysis of social media data can avoid false and misleading information to be spread, and it can also provide insight into general public attitude on a health-related issue (51). However, sentiment analyses sometimes fail to differentiate sufficiently, especially when it comes to comments containing long texts. To analyze such comments, methods that can distinguish positive from negative sentiment using sentiment analysis tools such as VADER (Valence Aware Dictionary and Sentiment Reasoner) are recommended (52). VADER is a rule-based dictionary model that can generate successful results with social media data, which can also score content based on its sentiment (41). One of the most important reasons for using the Vader method in sentiment analysis is its speed (53). VADER is highly effective for social media posts that contain characters such as emojis (54). Vader does not require a training dataset, and its results are interpretable (55). For this reason, the VADER method is generally preferred for sentiment analysis of social media text. VADER delivers its best performance with texts written in English (56). The texts in the studies are first translated into English, and then sentiment analysis is performed using VADER (57). VADER assigns scores in the range of (−1, 1) by considering both the intensity of the sentiment and the polarity of the words (56). Using sentiment analysis and the VADER tool to evaluate patient experiences shared on social media has provided valuable insights for managing intestinal diseases (58).

There are numerous publications in the literature that report on the outcomes of hemorrhoid treatment methods. Research on hemorrhoid treatment generally includes findings from randomized controlled trials comparing colorectal surgical methods (59–61). In addition to publications discussing the outcomes of surgical methods for the treatment of hemorrhoids, there are numerous publications detailing the outcomes of pharmacological treatment. These studies examine the effects of medications containing flavonoids and similar agents on hemorrhoids (62, 63). These experimental and observational studies include clinical findings on hemorrhoids. However, it does not provide information on the long-term effects of the treatment on patients. It does not include patients' opinions and emotions about their treatments. In the literature, studies that have conducted a societal assessment of hemorrhoids have generally been limited to the scale items used in the research. These studies generally examined awareness of hemorrhoids and the level of knowledge about them (64, 65). Studies in the literature have used traditional surveys and scales. These scales provide information only on a specific topic. There are very few studies in the literature that explore the public's attitudes and approaches toward hemorrhoids. Using infodemiology and text mining methods to discover information about the public's sentiments, thoughts, and attitudes regarding hemorrhoids will make significant contributions to the literature. A review of the existent literature shows that scientific studies that use sentiment analysis generally evaluate user comments on social media or e-commerce sites relying on positive or negative context. However, it is observed that the number of studies that examine differences or associations in societal attitudes by applying inferential methods on social media comments are few. It is also observed that most studies in the literature have examined social media comments or content written in English language.

Infodemiological research conducted in the field of proctology will greatly facilitate the management of hemorrhoids both before and after surgery. Analyzing data on hemorrhoids obtained from social media using text mining and infodemiological methods will enable researchers and physicians to easily access patients' views on hemorrhoid surgery. Researchers will no longer need to conduct field research to learn about patients' attitudes and opinions following hemorrhoid surgery.

In this study, text mining was used to evaluate Turkish patients' experiences and attitudes regarding hemorrhoid treatment options. The sentiment analysis of the comments regarding surgical, pharmacological, herbal, and traditional treatment methods were analyzed by the help of VADER tool. This process was followed by an analysis to compare the levels of benefit and satisfaction that each treatment methods provided.

In addition to examining social media comments on hemorrhoid treatment through sentiment analysis, the study examined word frequencies and relationships between words in the comments to draw conclusions related to infodemiological.

## 2 Material and methods

### 2.1 Data collection

The data source for our research consists of the comments written under Turkish-language YouTube videos related to hemorrhoids. For the process of obtaining comments, Python scripts were created using the YouTube Data API (66). During the selection of the videos, the videos containing the Turkish words “hemorrhoids” and “piles” were listed. Filtering was performed to determine videos with numerous comments related to hemorrhoids from among hundreds of videos. The videos that were included in the study did not have advertisements, did not promote commercial products, did not belong to drug or medical device companies, had a high number of views, and had at least 50 comments. To perform topic classification using the LDA method, videos with fewer than 50 comments were excluded from the analysis. The main reason for selecting a number of comments of 50 or more is that a sufficient number of comments is required to enable meaningful topic discovery using LDA. A small number of comments does not provide sufficient information for LDA modeling (41). Consequently, comments from 116 Turkish-language YouTube videos were included in the study. The total number of the comments included was determined to be 15,382.

The data included int the scope of the study was obtained from comments on videos posted on publicly accessible websites. The comments were kept fully anonymous, and no personal data was used. The data was obtained in total accordance with ethical regulations. Approval for the conduct of this study was obtained from the Medical Research Ethics Committee of Kahramanmaras Sutcu Imam University. (Protocol No: 2026/04-16; Approval Date: March 9, 2026).

### 2.2 Data Set

A dataset consisting of 15.382 comments under 116 Turkish YouTube videos related to “Hemorrhoids” was obtained. All comments were written in Turkish. However, the grammatical structure of the comments does not strictly follow Turkish grammar rules. There are a large number of emojis and punctuation marks within them. Some comments consist of very long words, while others were written only in a single word. The sentences were generally irregular and consisted of only a few words. The language mostly includes ironic and humorous words, as well as words used outside of their usual meaning, as would be expected from social media language. We investigated the most suitable sentiment analysis method for the dataset in our study. In structured sentences and long texts, BERT models perform better than the VADER method (67, 68). However, it was observed that the BERT model's performance lagged behind that of the VADER model in off-topic social media comments, health-related social media comments, and social media comments with irregular sentence structures (69–71). Therefore, it was predicted that conducting sentiment analysis on our dataset using only machine learning methods is would be insufficient for classification. It was observed by examining the knowledge within the literature that dictionary-based models would provide higher classification performance for comments collected from social platforms. For this reason, dictionary-bas ed models were preferred in our study. VADER is a powerful, dictionary-based sentiment analysis tool. VADER does not require a training dataset, unlike machine learning methods. VADER delivers fast results. Since Vader is rule-based, it does not take sentence structure or sentence order into account. For this reason, it delivers high performance in social media comments. Although VADER can perform sentiment analysis in various languages, it delivers its best performance in English. Because the Turkish language has an agglutinative structure, VADER may perform poorly on Turkish text (72). For this reason, Turkish comments were not used directly in our study. All Turkish comments were translated into English and analyzed using VADER. VADER tool was used to obtain sentiment scores for each comment. VADER is one of the models that has significantly high performance with social media language. It is specially designed for comments written in English, and it has been observed that it performs well in English comment analysis. To evaluate comments written in Turkish with the VADER model, these comments were translated into English. The relevant YouTube comments have been exported to an Excel file for the translation of the Turkish text into English. Emojis, URLs, and personal information have been removed from the comments in the Excel file. All comments were then translated into Turkish using Google Translate. To ensure the meaning is not lost in translation, a translator supervisor was consulted. The performance metrics for the Vader model are listed in Table 1. The performance of the VADER model in our study is consistent with the findings in the literature.

**Table 1:** Performance metrics of VADER model.

Sentiment	Precision	Recall	Accuracy	F1-Score	PPV	NPV
Negative	0,60	0,51	0,84	0,55	0,61	0,89
Neutral	0,70	0,84	0,72	0,76	0,70	0,77
Positive	0,72	0,51	0,81	0,60	0,72	0,83

### 2.2 Ethical considerations

The study was conducted in accordance with the Declaration of Helsinki, and approved by the Institutional Ethics Committee of Kahramanmaras Sutcu Imam University Medical Research Ethics Committee (protocol code 2026/04-16 and date March 09.03.2026) for studies involving humans.

### 2.3 Sentiment analysis and text mining process

For the sentiment analysis to be performed, text cleaning, and noise removal processes on each comment was performed. First, all words were converted to lowercase. Then, punctuation marks, symbols, emojis, and other similar words were removed out of the text. Following this, conjunctions and other similar words (stopwords) were eliminated. Numerical values, tags, and URLs are too frequently found in social media data. These tags and URLs were also removed until just words and raw text remain. Finally, tokenization was applied on the noise-free comments. The data preprocessing steps consist of the following stages.

- Comments containing advertisements, those posted for commercial purposes, and repetitive comments have been removed from the dataset.
- Translate; All comments in the dataset have been translated from Turkish to English.
- Lowercase Conversion: Words in the comments may contain uppercase or lowercase letters. All words in the dataset have been converted to lowercase by standard.
- Punctuation/Emoji cleaning; the dataset was obtained from social media posts. A large number of emojis and punctuation marks were used in the comments. At this stage, all emojis and punctuation marks were removed from the comments.
- Stopwords: Conjunctions and suffixes used in social media comments have been removed at this stage.
- Tag/Url/Character removing; All tags, special characters, web addresses, links, and special symbols have been completely removed from the comments.
- Tokenization/Lemmatization;Word tokenization was applied to long texts. Additionally, lemmatization was performed to remove affixes from the words and extract their roots.

The latent Dirichlet allocation (LDA) method was used to identify classes related to hemorrhoid treatment. LDA identifies hidden classes within text files. LDA takes into account the probability distributions of the words in the text and classifies text documents by topic based on these probabilities (73). LDA estimates the distribution of topics within each text and the distribution of words within those topics. In this way, it probabilistically classifies which words describe which topics (74). LDA is one of the unsupervised machine learning methods (75). Using the LDA method, the comments were classified into four different categories. These topics are classified as surgical, pharmacological, herbal, and traditional. A certain portion of the comments does not pertain to any specific topic. There are 5,710 comments in the surgical treatment category, 1,998 comments in the pharmacological treatment category, 781 comments in the herbal treatment category, and, finally, 114 comments in the traditional treatment category. Because the distributions of sample sizes across classes differed, nonparametric tests were used in the statistical analyses. A topic coherence analysis was conducted to evaluate the semantic consistency of the topics in the comments identified using the LDA method. The consistency coefficient was found to be 0.789. This coefficient indicates a high level of consistency.

Model selection was the first step to perform sentiment analysis. The dataset that was collected from social media comments. Then, a sentiment analysis was performed using a dictionary-based model, which was previously demonstrated successful results in classification. Next, by the VADER tool the sentiment scores of the comments were determined on a scale from −1 to 1. In this frame, scores close to −1 indicate negative sentiment, scores close to 1 indicate positive sentiment, and scores close to 0 indicate a neutral state. Figure 1 outlines the sentiment analysis processes in the study.

**Figure 1:**
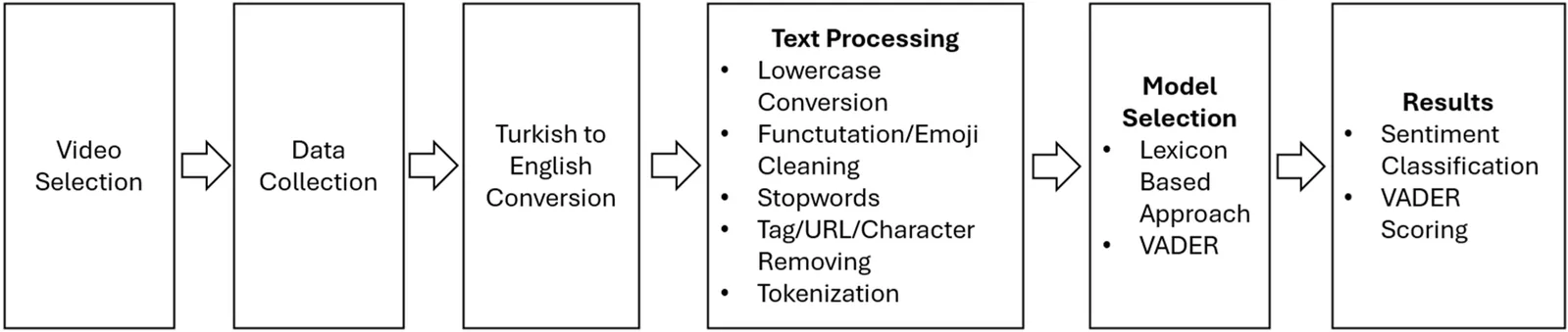
Sentiment analysis process.

Comments posted under videos related to hemorrhoids provide insight into societal attitude toward the hemorrhoids disease. Through the sentiment analysis methods, patients' treatment preferences and the protective measures they take against the disease were evaluated. For infodemiological research such as identification of the most frequently used medical terms, word frequencies and relationships between words were examined. All results have been supported by tables and graphs.

This study was designed as an infodemiological research project. Data cleaning, sentiment analysis, and text mining were analyzed using scripts created with the Spyder environment within the Python programming language. The study called on the Python programming language libraries NLTK, Pandas, Matplotlib, and VaderSentiment. A rule-based classification method was applied to the classification process. VADER sentiment scores were compared by statistical methods. The LDA (Latent Dirichlet Allocation) method was used to classify comments by topic.

### 2.4 Statistical analysis

The compliance of the quantitative data with the normal distribution was examined using the Kolmogorov–Smirnov test. For variables that did not conform to a normal distribution, comparisons between methods were examined using the Kruskal–Wallis H test. The Dunn-Sidak, one of the *post hoc* tests, was used to determine the source of the differences. To examine the distribution differences between the groups in relation to qualitative variables, the Chi-square test and Exact test were used. Statistical parameters were expressed as median (Q1–Q3), number (n), and percentage (%). The effect sizes are expressed as *η*^2^ and w. Statistical significance was accepted at *p* < 0.05. The data were evaluated using IBM SPSS version 22 (IBM SPSS for Windows version 22, IBM Corporation, Armonk, New York, United States) and Jamovi 2.6.44 software.

## 3 Results

The most frequently encountered unigram and bigram in the comments are shown in Table 2. Determining word frequencies provides important insights into the main themes of public opinion regarding hemorrhoids and helps identify societal trends. It is observed that the comments often include words related to health issues and ways to ease patient discomfort caused by hemorrhoids. Examination of the most frequently repeated bigram phrases indicates that they refer to diseases associated with hemorrhoids, protective measures, and different treatment methods. It is observed that the most frequently used words in the comments were “surgery,” “hemorrhoids,” and “pain”. It has been observed that surgical treatment, pharmacological treatment, and herbal treatment methods are frequently mentioned in individuals’ comments.

**Table 2:** Distribution of the most frequently used words in the comments.

Unigram				Bigram			
Words	*n*	Words	*n*	Words	*n*	Words	*n*
Surgery	2,358	Warm	370	Get well	1,020	Bleeding occurred	89
Pain	1,362	Drug	313	Had surgery	382	Pain occurred	79
Hemorrhoids	1,150	Nonsurgery	288	Post-surgery	158	Hemorrhoid surgery	77
Anus	1,077	Nodule	288	External hemorrhoids	136	Crohn's disease	67
Doctor	739	Constipation	281	Anal fissure	192	Definitive solution	64
Bleeding	690	Surgical	252	General surgery	121	Anal abscess	57
Suffer	557	Burning	221	Pain reliever	111	Rectal polyp	48
Cream	499	Anal	218	Anal disease	110	Rectocele causes	46
Laser	481	Proctology	203	Difficulty defecating	109	Anal swelling	45
Treatment	484	Defecation	173	Hot Water	108	Anal stenosis	44
Toilet	837	Itching	168	Constipation defecation	104	Laser surgery	40
Swelling	398	Suppository	161	Sit bath	103	Centaury oil	39
Blood	381			Shepherd's Purse	98		

To estimate the efficiency of the methods and plants used in hemorrhoid treatment, comments containing the words “beneficial” or “useless” alongside treatment methods and plant names were assessed. According to the findings in Table 3, the terms “surgery” and “beneficial” appeared together in 66.7% (*n* = 8) of the comments whereas the terms “medication” and “beneficial” appeared together in 47.1% (*n* = 16) of the comments. On the other hand, it was observed that the rate of comments mentioning shepherd's purse, St. John's wort, and bay leaf together was 100% (*n* = 8, *n* = 4, *n* = 6 respectively). The rate of the comments where the word “leech” and the word “useful” were used together was found to be 75% (*n* = 3).

**Table 3:** Frequency distributions of words used in conjunction with the terms “useful” and “not useful”.

Words	Useful	Not useful		Useful	Not useful
	*n* (%)	*n* (%)	Words	*n* (%)	*n* (%)
Drug	16 (47.1)	18 (52.9)	Black Cumin (*Nigella Sativa L.*)	2 (100.0)	0 (0.0)
Surgery	8 (66.7)	4 (33.3)	Garlic (*Allium sativum*)	2 (100.0)	0 (0.0)
Aubergine (*Solanum melongena L*.)	6 (66.7)	3 (33.3)	Horse Chestnut *(Aesculus hippocastanum)*	1 (100.0)	0 (0.0)
Shepherd's Purse (*Capsella bursa-pastoris*)	8 (100.0)	0 (0.0)	*Asphodelus Aestivus L.*	1 (100.0)	0 (0.0)
Bay Laurel Leaf *(Laurus nobilis L.)*	6 (100.0)	0 (0.0)	Dill (*Anethum graveolens*)	1 (100.0)	0 (0.0)
Fig leaf (*Ficus carica L*.)	4 (80.0)	1 (20.0)	Diet	1 (100.0)	0 (0.0)
St. John's Wort (*Hypericum Perforatum*)	4 (100.0)	0 (0.0)	Suppository	0 (0.0)	1 (100.0)
Nettle (*Urtica dioica*)	3 (75.0)	1 (25.0)	Melon (*Cucumis melo*)	1 (100.0)	0 (0.0)
Barberry (*Berberis Vulgaris*)	3 (75.0)	1 (25.0)	Thyme (*Thymus vulgaris L.)*	1 (100.0)	0 (0.0)
Leech Therapy	3 (75.0)	1 (25.0)	Linseed (*Linum usitatissimum*)	1 (100.0)	0 (0.0)
*Echinops ritro*	3 (100.0)	0 (0.0)	Cola	0 (0.0)	1 (100.0)
Cupping	2 (66.7)	1 (33.3)	Probiotic	1 (100.0)	0 (0,.0)
Laser	1 (33.3)	2 (66.7)	Fennel (*Foeniculum vulgare*)	1 (100.0)	0 (0,0)
Black-eyed herb (*Terminalia chebula*)	2 (100.0)	0 (0.0)	Butter	1 (100.0)	0 (0,0)
Pepper	0 (0.0)	2(100.0)	Turmeric (*Curcuma longa*)	1(100.0)	0(0,0)

In Table 4, words related to the four hemorrhoid treatment methods are grouped as surgical, pharmaceutical, herbal, and traditional. Table 4 also shows how often these words appear within the comments. As can be seen, the most frequently used word in comments regarding the surgical method was “surgery” whereas the word “cream” was first among the words in comments related to Pharmaceutical. The word “shepherd's purse” was the most consistent in comments related to herbal methods. Meanwhile, the word “leech” emerged as the most common term in comments concerning traditional treatment methods. Figure 2 illustrates these findings.

**Table 4:** Frequency distributions of the most frequently used terms by treatment method.

Surgical	*n*	Pharmaceutical	*n*	Herbal	*n*	Traditional	*n*
Surgery	2,358	Cream	499	Capsella bursa pastoris	129	Leech Therapy	89
Hemorrhoids	1,150	Drug	313	hypericum perforatum	63	Cupping	22
Anus	1,077	Suppository	161	Oliveoil	55		
Piles	506	Rectal Drug	240	Herbalist	55		
Laser	481	Pill	100	Fig	52		
Glands	283	Injection	100	Aubergine	45		
Surgical	252			Bay leaf	45		
Fistula	56			Apricot	40		
Botox	24			Black cumin	25		
				Nettle	25		

**Figure 2:**
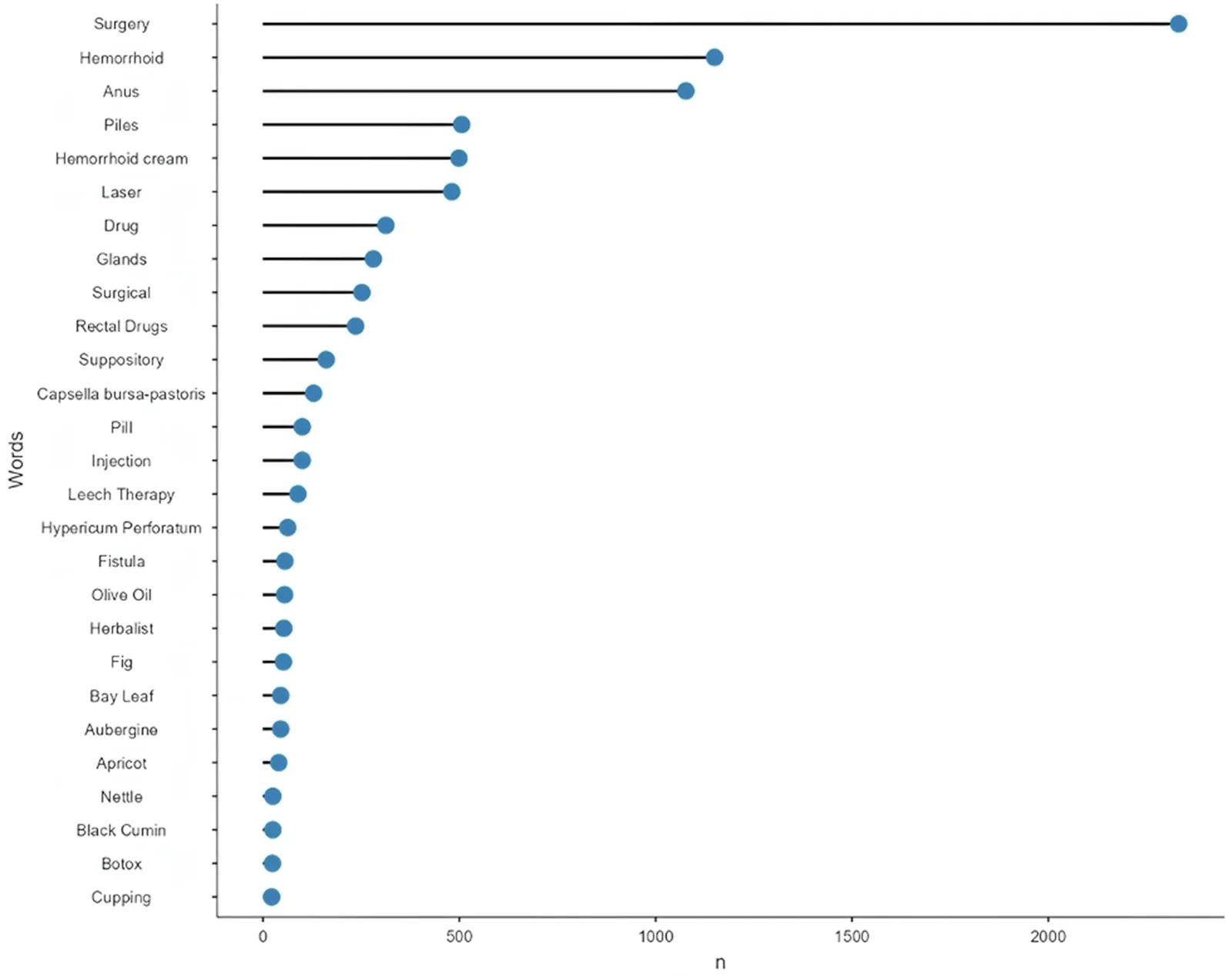
Frequency distributions of treatment-related terms in the comments.

In Table 5, the differences in the distribution of treatment methods used with the words “recover,” “ not recover,” and “recurrence” are shown. The distribution difference between surgical treatment methods and improvement status was found to be statistically significant (ES:0.584, *p* = 0.001). The percentage of comments that used words related to surgical treatment methods with the word “improved” was determined to be 34.1% (*n* = 15). The distribution difference between herbal treatment methods and improvement status was also found to be statistically notable (ES:0.523, *p* = 0.004). The percentage of comments using words related to herbal treatment methods with the word “improved” was obtained as 95.7% (*n* = 22). No distributional difference was found between traditional treatment methods and improvement status. Similarly, no distributional difference was found between pharmaceutical treatment and improvement status. The findings do not reflect the results of a clinical treatment for hemorrhoids. The findings represent the opinions of YouTube users regarding hemorrhoid treatment. These results are stated in Figure 3.

**Table 5:** Distribution of treatment methods by recovery status.

Treatment Methods	Disease					
	Not recover	Recover	Recurrence			
	*n* (%)	*n* (%)	*n* (%)	X^2^	ES (w)	*p*
Surgical	26 (59.1)	15 (34.1)	3 (6.8)	24.972	0,584	0.001^a^
Pharmaceutical	1 (33.3)	2 (66.7)	0 (0.0)	0.194	0,058	0.907
Herbal	1 (4.3)	22 (95.7)	0 (0.0)	19.985	0,523	0.004^a^
Traditional	0 (0.0)	3 (100.0)	0 (0.0)	2.309	0,177	0.315

Chi-Square test; Exact test; a:0.05.

a The distributional difference is statistically significant; ES(w):Effect size.

**Figure 3:**
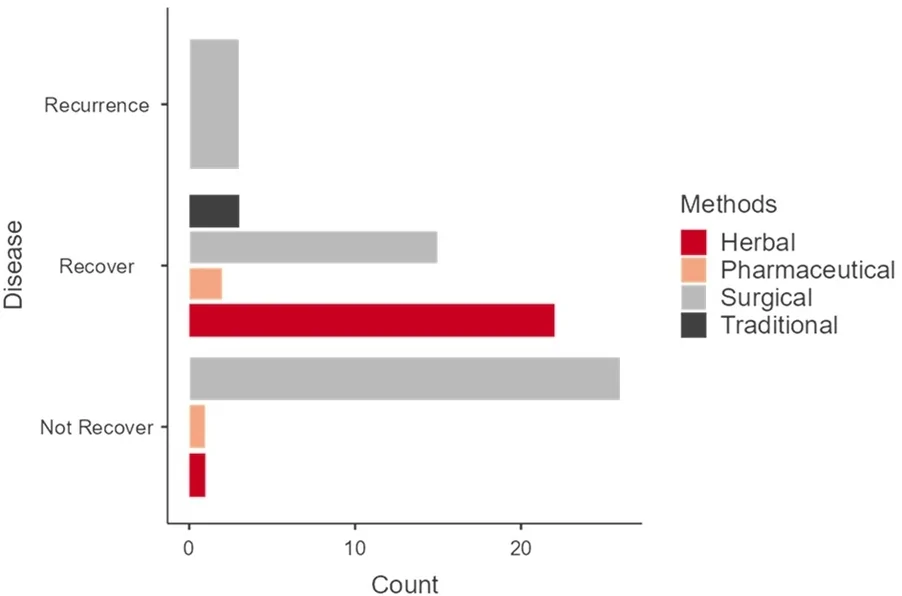
Distribution of treatment methods by recovery status.

In Table 6, the hemorrhoid treatment-related words frequently mentioned within comments are compared according to their VADER scores. High VADER scores indicate positive emotions, while low scores indicate negative emotions. Comments containing the words “surgery,” “suppository,” “blood letting,” “stinging nettle,” “St. John's wort oil,” “laser,” “rectal cream,” “garlic,” “leech,” and “olive oil” were compared according to their VADER scores. The differences in VADER scores between comments containing these words were found to be statistically significant (ES = 0.084, *p* < 0.001). Comments with the highest VADER scores contained the words “olive oil” and “St. John's wort oil.” Comments containing olive oil had statistically significantly higher VADER scores than comments containing “surgery”, “suppository”, “laser”, or “leech” (*p* < 0.05). Similarly, comments containing the word “St. John's wort” had statistically significantly higher VADER scores than comments containing the words “surgery,” “candle,” “laser,” and “leech” (*p* < 0.05).

**Table 6:** A comparison of VADER scores and sentiment for terms related to hemorrhoid treatment.

Words	Vader Score					Sentiment					
	Mean ± SD	Median (Min-Max)				Negative	Neutral	Positive			
			Statistics	*η* ^2^	*p*	*n* (%)	*n* (%)	*n* (%)	X^2^	ES(w)	*p*
Surgery	0.020 ± 0.244	0.000 (−0.981/0.923)	93.101	0.084	*p* < 0.001^a^	303 (11.2)	2,007 (74.4)	388 (14.4)	15.597	0.065	0.004^b^
Suppository	0.030 ± 0.287	0.000 (−0.951/0.927)				23 (13.5)	115 (67.3)	33 (19.3)	3.434	0.031	0.179
Cupping	0.128 ± 0.254	0.000 (−0.250/0.718)				1 (4.5)	15 (68.2)	6 (27.3)	2.808	0.028	0.245
Nettle (*Urtica dioica*)	0.145 ± 0.284	0.000 (0.000/0.840)				0 (0.0)	17 (77.3)	5 (22.7)	3.174	0.030	0.204
St. John's Wort ^c^^,^^d^^,^^e^^,^^f^ (*Hypericum Perforatum*)	0.166 ± 0.247	0.000 (−0.459/0.933)				2 (3.8)	25 (48.1)	25 (48.1)	42.357	0.110	0.001^b^
Laser	0.012 ± 0.261	0.000 (0.964/0.961)				40 (12.0)	244 (73.5)	48 (14.5)	0.746	0.014	0.688
Rectal Cream	0.077 ± 0.245	0.000 (−0.250/0.961)				4 (8.5)	34 (72.3)	9 (19.1)	0.633	0,013	0.728
Garlic (*Allium sativum*)	0.111 ± 0.273	0.000 (−0.250/0.459)				2 (16.7)	6 (50.0)	4 (33.3)	3.644	0.032	0.161
Leech Therapy	0.026 ± 0.160	0.000 (−0.250/0.840)				4 (5.8)	59 (85.5)	6 (8.7)	5.323	0.039	0.069
Olive Oil ^c^^,^^d^^,^^e^^,^^f^	0.204 ± 0.296	0.000 (−0.440/0.840)				2 (4.0)	27 (54.0)	21 (42.0)	27.250	0.088	0.001^b^

Kruskal Wallis h test;Post-hoc:Dunn Sidak test; Chi Square test;a:0.05.

a Difference between of groups is statistically significant.

b The distributional difference is statistically significant;.

c Difference compared to the surgery group is significant;.

d Difference compared to the suppository group is significant.

e The difference compared to the laser group is significant;.

f difference compared to the leech group is significant. ES(w):Effect size.

In the comments, distributional differences in sentiments (positive, negative, and neutral) were examined based on comments containing words commonly associated with hemorrhoid treatment. Statistically significant differences in the distribution of sentimental states were found in comments containing the word “surgery” (ES:0.065, *p* = 0.004). The rate of positive emotions in comments containing the word “surgery” was found to be 14.4%. Comments containing the word “St. John's wort” had a notable rate of positive emotions (ES:0.110, *p* = 0.001) with a rate of 48.1%. The difference was statistically significant also in comments containing the word “olive oil” (ES:0.088, *p* = 0.001). The rate of positive emotions was obtained as 42.0%.

In Table 7, the distribution of hemorrhoid treatment methods according to sentimental state was stated. The difference in distribution of sentimental states between comments containing words related to traditional treatment methods was found to be statistically significant (ES:0.048, *p* = 0.009). Comments containing traditional treatment methods had a positivity rate of 16.5% (*n* = 13). The difference in sentiment distribution among comments containing words related to surgical treatment methods was also found to be statistically significant (ES:0.082, *p* = 0.001). The positivity rate in comments containing words related to surgical methods was 14.7% (*n* = 437). The difference in distribution of sentimental states among comments containing words related to herbal treatment methods was also found to be statistically significant (ES:0.066, *p* = 0.001). The positivity rate of comments containing words related to herbal treatment methods was 20.9% (*n* = 232). No distributional difference in sentimental states was found for comments containing words related to pharmaceutical treatment methods (ES:0.031, *p* = 0.126). Figure 4 shows the mentioned results related to hemorrhoid treatment methods.

**Table 7:** Distribution of sentiment states by treatment method.

Treatment Methods	Sentiment Analysis					
	Negative	Neutral	Positive			
	*n* (%)	*n* (%)	*n* (%)	X^2^	ES(w)	*p*
Surgical	332 (11.2)	2,202 (74.1)	437 (14.7)	31.309	0.082	0.001^a^
Pharmaceutical	38 (8.4)	325 (71.9)	89 (19.7)	4.135	0.031	0.126
Herbal	94 (8.5)	785 (70.7)	232 (20.9)	20.249	0.066	0.001^a^
Traditional	0 (0.0)	66 (83.5)	13 (16.5)	9.256	0.048	0.009^a^

Chi-Square test; Exact test; a:0.05.

a The distributional difference is statistically significant; ES(w):Effect size.

**Figure 4:**
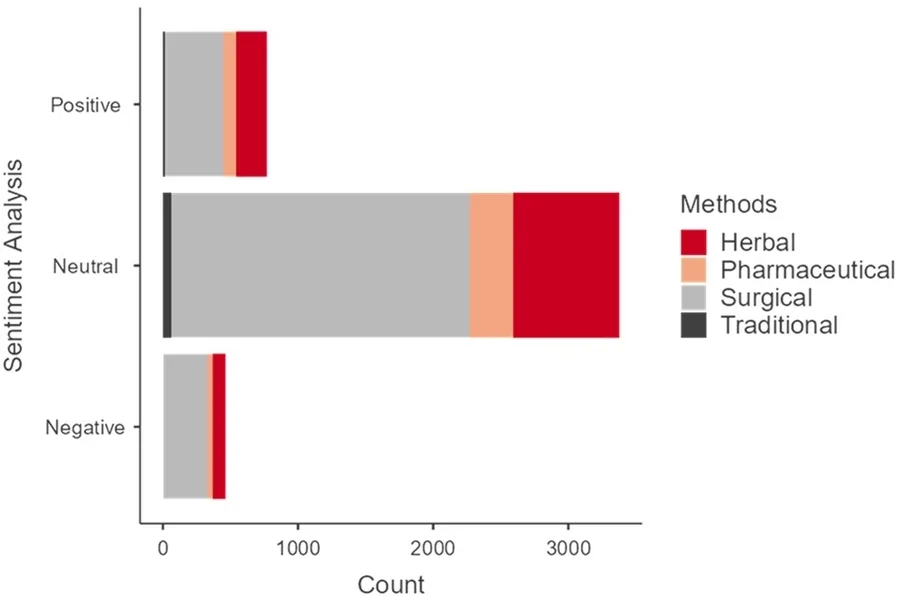
Distribution of Sentiment states by treatment method.

In Table 8, the VADER scores of comments regarding hemorrhoid treatment methods were compared. According to the findings, the score differences between comments containing hemorrhoid treatment methods was statistically significant (ES:0.041, *p* = 0.001). It was observed that the comments with the lowest VADER scores were those that included surgical treatment methods. Comments with the highest VADER scores included traditional treatment methods. These findings are shown in Figure 5.

**Table 8:** Comparison of VADER scores by treatment method.

Treatment Methods	VADER Score				
	Mean ± SD	Median (Min/Max)	Statistics	ES (η^2^)	*p*
Traditional	0.082 ± 0.202	0.000 (0.000/0.952)	43.873	0.041	0.001^d^
Surgical^a^^,^^b^^,^^c^	0.022 ± 0.244	0.000(−0.981/0.961)			
Herbal	0.069 ± 0.260	0.000(−0.951/0.981)			
Pharmaceutical	0.058 ± 0.248	0.000(−0.883/0.900)			

Kruskal Wallis h test; Post-hoc:Dunn Sidak test; *α*:0.05.

a The difference is significant compared to traditional medical methods.

b The difference is significant compared to herbal methods.

c The difference is significant compared to pharmaceutical methods.

d difference between of groups is statistically significant.; ES(η^2^): Effect size.

**Figure 5:**
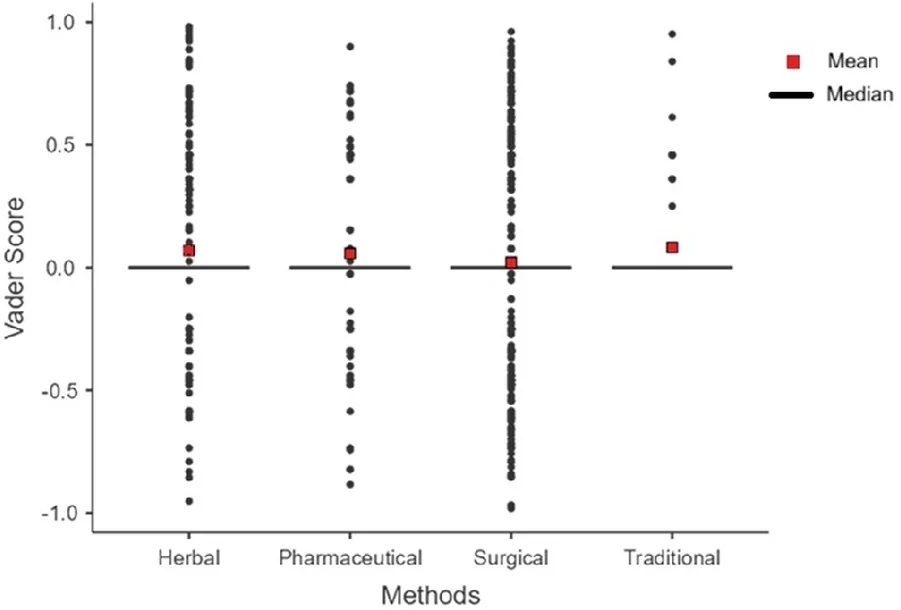
Comparison of VADER scores by treatment method.

## 4 Discussion

Text mining methods and infodemiological research provide important insights into how societies approach health-related issues. The rapid increase in social media usage and proliferation of websites that allow users to add content and comments have created large data sets. Text mining and infodemiological methods can be used to discover information from big data sets. Infodemiological science examines the dissemination of public health information in digital environments, such as the internet, and its effects on society (45). This method has frequently been used in studies conducted during the Covid-19 pandemic period. Many infodemiological studies have examined the spread of the Covid-19 disease and how society has responded to it (76–79).

In our study, an in-depth analysis of social media comments was conducted for the purpose of evaluating Turkish society's attitudes, feelings, treatment preferences, and existent knowledge regarding hemorrhoids, using sentiment analysis and text mining methods.

The most commonly used single-word (unigram) and two-word (bigram) terms were identified within the comments. These terms were then used to conclude on Turkish society's approach to hemorrhoids. The existence of words such as “surgery,” “pain,” and “bleeding” provided valuable information about the frequency of surgical treatment and the probability of pain or bleeding complaints. Examination on the most commonly used words revealed that they were mostly related to treatment types and symptoms.

To understand if individuals benefited from hemorrhoid treatment or not, the most commonly used words alongside “beneficial” and “useless” were identified within the comments. The words that were mostly used alongside “beneficial” were “Shepherd's Pouch,” “Bay Laurel Leaf,” and “St. John's Wort.” The fact that all three of these words are related to herbal treatments clearly indicates that people benefit from herbal treatments. Said et al. (80) tested a topical cream containing several plant extracts on patients with hemorrhoids in a randomized controlled trial. By the end of day 6, the cream successfully reduced pain intensity and itching in these patients from grade 2 to grade 0 and grade 1. In the experimental group treated with the herbal cream, pain was significantly reduced compared to the control group (80). Zisis et al. (34) investigated the efficacy of the Arum maculatum plant in the treatment of symptomatic hemorrhoids. A significantly more successful recovery was observed in the treatment group that used Arum maculatum. Significant improvements in patients' quality of life were observed (34). The information within the literature supports our findings. It was reported in many studies that herbal treatment is widely used to treat hemorrhoids (35, 81, 82). In contrast, improvements achieved through herbal treatment are generally symptomatic and temporary. A complete cure is possible with surgical treatment. However, herbal treatment only alleviates pain, itching, and bleeding.

Sentiment analysis findings reveal that the general public has neutral feelings about hemorrhoid treatment methods. However, the distribution of public opinion on treatment methods showed statistically significant differences. Sentiments towards herbal methods were observed to be proportionally more positive than those towards other methods. The ease of application, high accessibility, low cost, and ability to provide results quickly play an important role in the positive feelings associated with herbal treatment. In their study, Emin et al. (83) investigated the efficacy of the plant Teucrium orientale L. in treating hemorrhoids. Approximately 90% of users report relief from bleeding, pain, and itching associated with hemorrhoids. Herbs may be effective in reducing symptoms, but they do not provide a complete cure (83). Positive sentiments towards surgical treatment methods were lower than those towards other methods. The main reasons for this were the painful and uncomfortable post-operative process. In hemorrhoid surgery, pain levels peak during the first 24–48 h. This period can sometimes last up to one week (84). The rate of moderate pain following excisional surgery is reported to be 65% (85). The absence of negative comments about traditional treatment methods does not stem from satisfaction with the treatment but stems from the small number of comments about these methods. Traditional treatment methods are generally used during the postoperative period. This treatment can be effective in reducing postoperative pain and bleeding. In their study, Duan et al. (86) applied traditional Chinese medicine to patients with postoperative hemorrhoids. As a result, the treatment group had pain and edema scores that were approximately 25% lower than those of the control group (86). Similar to traditional treatment, there have been very few comments regarding sclerotherapy. These comments did not include any positive or negative assessments of sclerotherapy. Sclerotherapy is generally preferred for Stage I and Stage III hemorrhoids (33). In a meta-analysis conducted by Gallo et al. (25), it is stated that very high success rates were achieved with sclerotherapy (25).

The VADER score is a valuable tool for quantitative comparison of sentiments related to comments. Our evaluation using VADER scores revealed differences in sentiment scores between treatment methods. In general, all hemorrhoid treatment methods received scores close to 0, indicating a neutral emotional state. However, when the treatment methods were compared, traditional, herbal, and medical drug treatments were observed to have higher positive emotion scores than surgical treatments. The low VADER sentiment scores for surgical treatment are related to the post-operative pain and discomfort (84). On the other hand, traditional treatment methods have the highest VADER emotion scores. These scores stem from the capability of cupping and leech therapy to remove clotted blood in the anal region, thus reduce swelling. Individual satisfaction levels according to treatment method are aligned with the literature. In their research, Quan et al. (10) state that, although surgical methods provide faster recovery and lower recurrence rates in hemorrhoid treatment, protective treatment methods are preferred for their safety and comfort (10). Surgical methods are the most effective treatments for hemorrhoids. Herbal, pharmaceutical, and traditional treatment methods help alleviate symptoms during the postoperative period. Miga-Orcyzkowska et al. (87) stated that non-surgical methods significantly contribute to the treatment of hemorrhoids when compared to surgical methods (87). The findings in our study show that comments on herbal treatment methods for hemorrhoids are more positive than comments on pharmaceutical treatment methods.

One important finding in our study is the evaluation of comments demonstrating recovery rates according to treatment method. While comments regarding traditional treatment methods often mention improvement, comments regarding surgical treatment methods include reports that the hemorrhoids have recurred. This result is not consistent with the literature. The literature indicates that surgical methods are the most effective treatment for hemorrhoids and that the lowest recurrence rate is achieved with surgical methods (88). Zgriadskii et al. (89) stated that conservative methods yielded good results for stages I and II hemorrhoids and that surgical methods reduced recurrence and provided effective treatment for advanced stages (89).

In this study, the VADER scores and statistical distributions of sentiment for the most frequently used words related to hemorrhoid treatment were compared. The highest VADER scores were observed for plants used in herbal treatments. “Olive oil” had the highest score, followed by “St. John's wort” and “stinging nettle.” It is observed that the words with the lowest scores were “surgery” and “laser”. Examining the distribution of sentiment states according to the most frequently used words in the comments revealed statistically significant differences among the words “surgery,” “St. John's wort,” and “olive oil.” The percentage of positive emotions associated with “surgery” is low, while the percentages associated with “St. John's wort” and “olive oil” are higher. The reason the VADER score is low for the term “laser” is that, as with surgical treatment, it stems from postoperative pain in laser treatment as well. The possibility of experiencing pain and that the disease will recur after laser treatment has led to the view that laser treatment does not significantly differ from surgical methods in terms of post-operative comfort. Wee et al. (90) stated in their study that the probability of recurrence after traditional surgical and laser treatment methods were similar (90). The main reason for patients' positive views on herbal treatments is thought to be that they provide temporary, effortless, and rapid relief from the pain and discomfort caused by hemorrhoids. Additionally, herbal methods are thought to contribute to higher positive sentiment rates due to their lower cost and greater accessibility compared to surgical methods. There are many studies in which herbal treatments have been used. In their study, Hashempur et al. (91) included St. John's wort among the medicinal plants used to treat hemorrhoids (91). Caliskan et al. (92) stated that St. John's wort was used to treat hemorrhoids in Anatolia (92). The fact that herbal or traditional treatments yield positive emotional scores does not necessarily reflect treatment success. The most effective methods for treating hemorrhoids are surgical procedures. Other methods consist of treatments aimed at alleviating symptoms.

The findings from this study provide important insights for evaluating the outcomes of surgical techniques used in hemorrhoid surgery and for improving treatment methods. Sentiment analysis provides insights into public perceptions regarding pain management following surgical treatment, disease recurrence management, and quality of life after surgery. The findings of sentiment analysis help improve quality of life in patients with hemorrhoids, shorten postoperative recovery times, evaluate alternatives and community-based approaches in the context of preventive medicine, discuss treatment methods, and provide a detailed examination of the factors contributing to the development of the disease. Information gathered from social media comments can be used to draw conclusions about patients' levels of knowledge. Patients can be provided with accurate health information. Patients can be educated about hemorrhoids.

There are numerous comments on social media describing experiences with surgical procedures. This information is important for assessing long-term and short-term postoperative outcomes. There were comments from patients who had undergone multiple surgical procedures. Some patients who did not have a successful outcome with the De Longo method or the laser method stated that conventional hemorrhoidectomy was the best solution. Patients have stated that open and closed hemorrhoidectomy is the best method in terms of preventing recurrence. Patients report that they are satisfied with the Band ligation method in terms of postoperative pain management. Some patients have reported that the band ligation method causes narrowing of the anus. There are numerous comments regarding laser surgery. Patients have indicated that they are bothered by postoperative pain resulting from the laser surgery. There are numerous comments regarding laser surgery. Patients have reported experiencing postoperative pain following laser surgery. Some patients have indicated that they would like to try different treatment methods. Some patients who were not successful with the De Longo and laser methods have expressed interest in the hemorrhoidal artery ligation method.

Our study was conducted using social media data. When working with social media data, there are certain points to keep in focus. There are many YouTube channels created for advertising purposes. Videos from these channels may contain biased comments. For this reason, videos intended for advertising or commercial purposes, as well as comments related to these videos, have been disabled. The comments were reviewed and selected with great care. Comments related to videos by healthcare professionals and healthcare organizations were selected. It should also be noted that while social media comments can be beneficial to people, they can also lead to an infodemic. Sharing misinformation about health on social media can cause serious harm to public health. Therefore, seeking information from experts in the field of health can help prevent the spread of inaccurate and misleading information.

In terms of social media data, sampling bias poses a risk (93). By including all comments that meet the study's criteria, sample bias can be eliminated.

### 4.1 Limitations

There were a large number of videos on YouTube related to hemorrhoids. However, most of these videos had very few comments. To enable evaluation using LDA, comments from videos with 50 or more comments were included in the study. Since the study aimed to investigate the Turkish Society's attitudes toward hemorrhoids, comments in other languages were not included in the study. The findings of the study do not compare the effectiveness of hemorrhoid treatments. Instead, they compare the public's attitudes and perceptions regarding hemorrhoid treatments. Another important limitation of the study stems from the absence of verification of social media comments against clinical diagnoses, disease stage, and patient clinical data. Social media comments may contain subjective comments. These comments reflect individuals' experiences and subjective opinions rather than medically measurable results.

## 5 Conclusion

In this study comments under Turkish-language YouTube videos concerning hemorrhoids were assessed. The treatment method preferences and sentiments of YouTube users regarding the disease were comparatively analyzed. It was observed that users had more positive sentiments about herbal and traditional medicine methods when compared to surgical methods. Although surgical methods are the only treatment that can offer a definitive solution for hemorrhoids, symptoms such as pain, bleeding, and itching experienced during the postoperative period diminish patients' positive perceptions. The positive sentiments towards herbal and traditional treatment methods are partly due to the fact that these methods temporarily alleviate hemorrhoid symptoms.

It was observed that users most frequently mentioned words related to surgical treatment methods and disease symptoms in the comments. Sentiment analysis and text mining methods are highly successful techniques for determining public attitudes toward hemorrhoids.

Text mining and infodemiology methods provide the physician with information on important issues such as complications arising after surgery, ongoing or resolved symptoms, postoperative medications, and the course of pain. By evaluating this information and feedback, proctology specialists can further improve the surgical procedure.

This study focuses exclusively on comments regarding hemorrhoids made by the Turkish public on social media. Researchers in the field of health should make greater use of social media data in accordance with scientific standards. We recommend that researchers interested in this topic conduct a comprehensive study of all societies and include comments on hemorrhoids in all languages in their research. We also recommend that researchers include not only YouTube but all social media platforms and websites containing patient reviews in their research.

## Data Availability

The original contributions presented in the study are included in the article/Supplementary Material, further inquiries can be directed to the corresponding author/s.
